# Supplementary material for: Knowledge about HPV Infection and the HPV Vaccine among Parents in Southeastern Serbia
Source: Medicina (Kaunas). 2022 Nov 22;58(12):1697. doi: 10.3390/medicina58121697 (PMC9785943; doi:10.3390/medicina58121697)
Supplement: Supplementary file 1 [file medicina-58-01697-s001.zip › medicina-1953739-supplementary.pdf]

Supplement File S1

Anonymous questionnaire about knowledge about HPV infection and the HPV vaccine

I Section

1. Sex males females
2. Age (in years)___________________________________
3. Place of residence_______________________________
4. Type of education: medical non-medical
5. Pediatrician recommendation Yes No

II Section-Knowledge about HPV infection

6. How can you get infected with HPV?_____________________________
7. What are the main ways of transmission?_________________________
8. Who is at higher risk of HPV infection?
   Men
   Women
   I don't know
9. Are the HPV infections and malignant disease connected?
   Yes
   No
   I don't know
10. Does HPV infection always lead to the clinical manifestation of the disease?
    Yes
    No
    I don't know
11. Do you know what is the Pap smear test ?
    Yes
    No
    I don't know
12. What factors increase the risk of developing cervical cancer?
13. How can HPV infection be prevented?
    By vaccine and by condom
    masks
    I don't know

II Section- Knowledge about HPV vaccine

14. Is there a vaccine against HPV infection?
    Yes
    No
    I don t know
15. Is the HPV vaccine available in Serbia?
    Yes
    No
    I don t know
16. When is the best time to administrate the HPV vaccine?
    In children 9-14
    In children 15-19
    I don t know
17. Is the HPV safe?

Yes

No

I don t know

18. Are You worried about multiple doses of the HPV vaccine?

Yes

No

I don t know

19. What are the main side effects of HPV vaccine?

Yes

No

I don t know

20. Is there are a long life protection after HPV vaccination?

Yes

No

I don t know
